# Microarrayed Jug r 1 shows the best diagnostic performance in walnut allergy

**DOI:** 10.1186/2045-7022-5-S3-P20

**Published:** 2015-03-30

**Authors:** Maria Pedrosa, Teresa Boyano-Martínez, Carmen García-Ara, Teresa Caballero, Santiago Quirce

**Affiliations:** 1 Hospital La Paz Institute for Health Research (IdiPAZ), Madrid, Spain

## 

Several walnut (WN) allergens have been identified to date: Jug r 1 (2S albumin), Jug r 2 (7S globulin), Jug r 3 (LTP), Jug r 4 (11S globulin) and Jug r 5 (profilin), but their clinical significance is still to be determined in some cases. Allergen microarray (ISAC) provides information for three of these allergens.

The aim of the study was to determine the importance of component resolved diagnosis in WN allergic patients.

Ninety children suspected to have allergy to fruit, nuts and/or legumes were selected. Patients were classified as allergic if they presented at least 2 reactions unequivocally related to WN ingestion in the last 2 years. Patients were defined as tolerant if they consumed WN on a regular basis. Clinical questionnaire, skin prick test (SPT), total and specific IgE and ImmunoCAP ISAC (Thermo Fisher) were performed.

**Figure 1:**
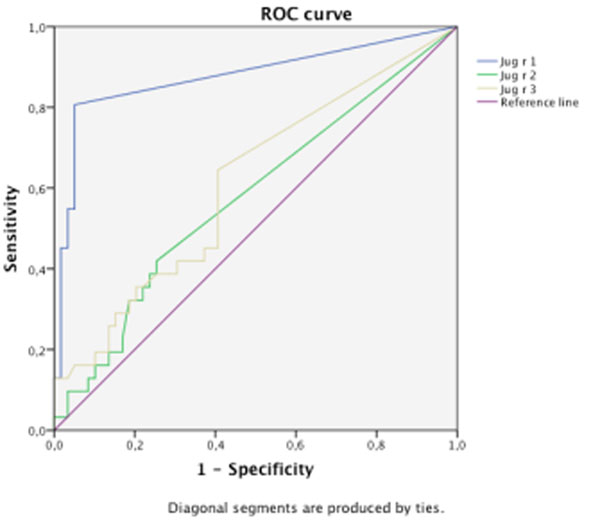
ROC curves for Walnut allergens

Thirty-one patients (18 males) were defined as allergic and 59 (36 males) tolerant. WN-SPT wheal size (median diameter 5.25 mm; IQR: 4-7 vs. 0 mm; IQR: 0-2, p=0.000) and WN-sIgE (median 5.35 kU/L; IQR: 3.52-12.84 vs. 0.65 kU/L; IQR: 0.08-1.86, p=0.000) were significantly greater in allergic than in tolerant children. Positive Jug r 1-sIgE was significantly more frequent in allergic patients (80.65% vs. 5.08%, p=0.000). Jug r 1-sIgE values were also significantly higher in allergic children (median 2.5 ISU; IQR: 0-7.6 vs. 0 ISU; IQR: 0-0, p=0.000). This was not found for Jug r 2 or Jug r 3. ROC curves were constructed for the three allergens showing Jug r 1 the best diagnostic performance (AUC: 0.876, 95%CI: 0.788-0.965, p=0.000) (Figure 1 and Table 1).

**Table 1:** Diagnostic performance of walnut allergens (MAISAC)

	Jug r 1	Jug r 2	Jug r 3
Sensitivity	0.81	0.42	0.65

Specificity	0.95	0.75	0.59

PPV	0,89	0.46	0.45

NPV	0.90	0.71	0.76

LR test +	15.86	1.65	1.59

LR test -	0.20	0.78	0.60

Post Test + Probability	0.89	0.46	0.45

Post Test - Probability	0.10	0.29	0.24

In conclusion, Jug r 1-sIgE is the best discriminating allergen in WN allergic patients.

